# MicroRNA profiles of four induced pluripotent stem cell lines derived from distinct tissues

**DOI:** 10.1186/s13104-025-07437-3

**Published:** 2025-08-17

**Authors:** Kongtana Trakarnsanga, Nutchavadee Vorasan, Nutnicha Tantarungsee, Methichit Wattanapanitch, Chinnavuth Vatanashevanopakorn, Bhoom Suktitipat, Chanatip Metheetrairut

**Affiliations:** 1 Department of Biochemistry, Faculty of Medicine Siriraj Hospital, Mahidol University, Bangkok, Thailand; 2 Genetic Epidemiology Research Group, Research Division, Faculty of Medicine Siriraj Hospital, Mahidol University, Bangkok, Thailand; 3 Siriraj Center for Regenerative Medicine, Faculty of Medicine Siriraj Hospital, Mahidol University, Bangkok, Thailand; 4 Integrative Computational Bioscience Center, Mahidol University, Nakhon Pathom, Thailand

**Keywords:** Induced pluripotent stem cells, Epigenetic profiles, microRNA profiles

## Abstract

**Objective:**

Induced pluripotent stem cells (iPSCs) are generated from a vast number of adult cell types. While they all acquired embryonic stem cell (ESC)-like properties during reprogramming, differences in certain characteristics, including differentiation potential, remained. These differences are hypothesized to be due to epigenetic memory or individual genetic background.

**Results:**

This study compared microRNA (miRNA) profiles, which is one aspect of epigenetic regulation, of four iPSC lines derived from dermal fibroblasts, hair follicle keratinocytes, peripheral blood T cells, and exfoliated renal epithelial cells by nCounter^®^ miRNA expression panels. 110 miRNAs were expressed in all iPSC lines, which accounted for 67.5–75.9% of expressed miRNAs in each line, while there were only 3, 4, 9, and 28 uniquely expressed miRNAs in each line. MiRNA profiles of the four iPSC lines clustered together, but away from those of mature cells of origin or ESCs retrieved from the Gene Expression Omnibus database as analyzed by principal component analysis. These results suggested that the miRNA profiles of the four iPSC lines are mostly similar to each other, though with some specific unique gene expression, and do not appear to indicate any obvious differences between the iPSC lines derived from different adult cell types in terms of miRNA expression.

**Supplementary Information:**

The online version contains supplementary material available at 10.1186/s13104-025-07437-3.

## Introduction

A vast number of adult cell types have been shown to be viable sources of reprogramming to generate iPSCs [1, 2]. While iPSC lines have each been demonstrated to possess ESC-like properties, several studies still observed certain different characteristics and varying differentiation potential among iPSC lines [2–8]. One study on fibroblast-derived and blood-derived iPSCs showed that iPSCs tended to differentiate more readily into related cell types, while the differentiation efficiency was lower when they were directed towards less related cell types [5]. In contrast, another study found similar erythroid differentiation potential from CD34^+^ hematopoietic stem cells-derived iPSCs, neural stem cells-derived iPSCs, and fibroblast-derived iPSCs [9]. Furthermore, another study demonstrated that iPSC differentiation potential is dependent on the expression of a specific gene, IGF2, and the methylation pattern regardless of the cells of origin [6]. Moreover, another study demonstrated that as iPSCs were maintained in culture for longer, their differentiation potential became less varied [7, 10].

The differences between iPSC lines generated from different tissues are believed to be due to epigenetic memory, although this issue is still under debate [2, 3, 5–9, 11–13]. For example, as opposed to epigenetic memory, previous studies argued that iPSC heterogeneity resulted from their diverse genetic background [11, 14, 15]. Several studies have focused on the differences in DNA methylation patterns between ESCs and iPSCs, between iPSCs and their somatic donor cells, and among iPSC lines [2–8, 11–13]. In addition, some studies observed differences in posttranslational covalent histone modifications among iPSC lines [12, 16]. In addition to DNA methylation and histone modifications, microRNA (miRNA) is also considered part of epigenetic regulation as they function in regulating gene expression [10, 17–20]. Previous studies have found significant differences in miRNA profiles of ESCs and iPSCs [16, 21, 22]. In addition, a study compared miRNA profiles of cord blood-derived vs. fibroblast-derived iPSCs, and identified *hsa*-miR-155 as a factor that promoted the differentiation of cord blood-derived iPSCs to hematopoietic progenitor cells [10]. However, to date there is no study that compares miRNA profiles of several iPSC lines generated from different tissues of distinct lineages in humans.

This study compared miRNA profiles of 4 iPSC lines that were derived from dermal fibroblasts, hair follicle keratinocytes, peripheral blood T cells, and exfoliated renal epithelial cells [23–26] by copy number count method (nCounter® miRNA expression assay) and determined their similarity with PCA and miRNA target pathway analyses. We found that the miRNA profiles of the four iPSC lines were generally similar to each other, though with a few specific unique gene expression, rather than similar to each mature cell of origin or to embryonic stem cells.

## Methods

### Culture of iPSCs

The following iPSC lines were used in this study: MUSIi001-A (SF) [23], MUSIi006-A (SK) [24], MUSIi011-A (ST) [25], and MUSIi005-A (SU) [26]. All iPSC lines were cultured on Matrigel-coated wells (Matrigel (BD Biosciences) in Essential 8 medium (Gibco)) at 37 °C with 5% CO_2_. The medium was half-changed every day. Subculturing was performed by the addition of 50 mM EDTA (Gibco).

### RNA extraction

The harvested cells were lysed by QIAzol Lysis Reagent (Qiagen) and stored at −80 °C until RNA extraction. RNA extraction was performed according to the manufacturer’s protocol of the miRNeasy mini kit (Qiagen). Briefly, RNA was extracted by a phenol/chloroform-based method, followed by a silica-membrane-based purification in the RNeasy Mini spin column. Total RNAs were eluted in RNase-free water. RNA concentration and purity were determined by a nanophotometer (Implen nanophotometer P300).

### Quantitative real-time polymerase chain reaction (qRT-PCR)

Reverse transcription was performed according to the manufacturer’s protocol of miScript II RT Kit (Qiagen), followed by real-time PCR reaction according to the protocol of miScript SYBR^®^ Green PCR Kit (Qiagen). qRT-PCR was performed in triplicates and with the miScript Primer Assays: Hs_RNU6-2_11, Hs_miR-16_2, Hs_miR-145-5p, and Hs_miR-302a-3p (Qiagen). Real-time PCR reactions were performed on the LightCycler^®^ 480 Instrument (Roche).

### miRNA expression profile analysis

miRNA expression profile of each iPSC line was examined by the nCounter^®^ miRNA expression assay (Human v3 miRNA panel) on the nCounter^®^ Analysis System instruments according to the manufacturer’s protocol (NanoString Technologies) (see Additional file 1). Reporter code counts (RCC) data files were analyzed by the nSolver^™^ 4.0 Analysis Software (NanoString Technologies: https://nanostring.com/products/ncounter-analysis-system/ncounter-analysis-solutions/). Initially, quality control (QC) parameters for all samples: Imaging QC, Binding Density QC, Positive Control Linearity QC, Positive Control Limit of Detection QC, and Ligation QC analyses were examined. Background subtraction was carried out with the geometric mean of all negative control counts. Counts were normalized first with the geometric mean of positive control counts and then with the geometric mean of the top 100 expressed genes. Normalized counts were used for further analysis. “Expressed miRNAs” were defined to be those with normalized counts above the 95% confidence interval (arithmetic mean + 1.96 S.D.) of normalized counts of negative controls.

Scatter plots and the R-squared values from linear regression of each pair of comparison were created by the nSolver™ 4.0 Analysis Software (NanoString Technologies). Normalized counts were used to create scatter plots which encompassed individual data points assigned to Cartesian coordinates, and each iPSC line was represented by each axis of that specific pair of comparison.

### NanoString miRNA expression data from Gene Expression Omnibus

NanoString reporter code counts of normal fibroblasts, keratinocytes, renal epithelial cells, T lymphocytes, and ESCs were retrieved from the Gene Expression Omnibus (GEO) database. Data normalization by NanoStringNorm package [27] was described in Additional file 1.

### Statistical analysis

Principal component analyses (PCAs) were performed in the R statistical language. Normalized counts with no variations were excluded from the analysis.

Ct values from real-time PCR reactions were analyzed by the delta-delta Ct method [28] and two-tailed Student’s *t*-test between comparison pairs or Kruskal–Wallis Test for the comparison of four groups.

Pathway analyses of microRNA target genes were performed by miRPathDB v2.0 (https://mpd.bioinf.uni-sb.de/), which catalogs miRNA-target interactions (MTIs) and signaling pathways from several resources [29]. Here we selected (1) MTIs with strong experimental evidence, or (2) MTIs with any experimental evidence if (1) did not exist. From these targets, the miRPathDB v2.0 algorithm determined enriched pathways by a hypergeometric test [29]. The custom heatmap calculator function was utilized to generate the heatmap showing enriched pathways in the Gene Ontology-Biological Processes database.

## Results and discussion

### RNA samples from iPSC lines

In order to study microRNA profiles as one of the epigenetic characteristics of iPSCs generated from distinct human cell types, the 4 iPSC lines chosen for this study were generated from 4 different mature cell types of different lineages (Table 1). They were all derived from female subjects and were induced to become pluripotent stem cells by using integration-free Sendai viral vector to deliver the reprogramming factors: *OCT3/4*, *SOX2*, *KLF4*, and *c-MYC* [23–26]. The morphology of all iPSC lines (passage 35) right before they were collected for RNA extraction was similar to each other and to those in the initially published articles [23–26] (Fig. S1). The RNA expression level of a housekeeper gene, *RNU6B*, was shown by qRT-PCR to be expressed at similar levels in all samples (*p* = 0.063) (Fig. S2).

**Table 1:** The iPSC lines used in this study

iPSCs	Cell types of origin	Cell lineages
MUSIi001-A (SF)	Dermal fibroblasts from a cesarean section scar	Lateral plate mesoderm [30]
MUSIi006-A (SK)	Hair follicle keratinocytes	Ectoderm [31]
MUSIi011-A (ST)	T lymphocytes	Mesoderm [32]
MUSIi005-A (SU)	Exfoliated epithelial cells in urine (renal epithelial cells and transitional epithelial cells)	Intermediate mesoderm, endoderm [33–35]

**Fig. 1:**
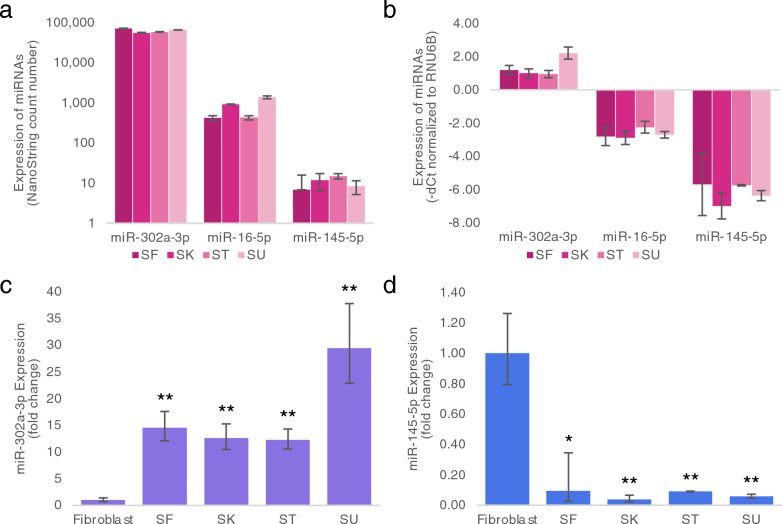
Comparison of expression of specific miRNAs. **a** Normalized count numbers (n = 3) of miR-302a-3p, miR-16-5p, and miR-145-5p as measured by nCounter^®^ miRNA expression assay. **b** Expression levels (n = 3), shown as -dCT normalized to RNU6B, of miR-302a-3p, miR-16-5p, and miR-145-5p as measured by qRT-PCR. Expression levels of **c** miR-302a-3p and **d** miR-145-5p in fibroblasts and the SF, SK, ST, and SU iPSC lines as measured by qRT-PCR (n = 3) and shown here as fold changes when normalized to those in fibroblasts. *p < 0.05, **p < 0.001, as analyzed by two-tailed Student’s *t*-test. Error bars designate standard deviations

Representative miRNAs were also examined by qRT-PCR and compared to their NanoString normalized count numbers. We found that the stem cell-specific miR-302a-3p was highly expressed in all 4 iPSC lines, whereas the differentiation specific miR-145-5p was expressed very lowly in all 4 iPSC lines when measured by both methods (Fig. 1a–d). In addition, when measuring by both methods, the expression of miR-16-5p was at moderate levels (Fig. 1a, b). These findings are similar to what have been reported by a previous study [22]. We also confirmed that all 4 iPSC lines expressed miR-302a-3p at significantly higher levels than that in fibroblasts (p < 0.001 for all iPSC lines) while all iPSC lines expressed miR-145-5p at significantly lower levels than that in fibroblasts (p < 0.001 for all iPSC lines) (Fig. 1c, d).

### MicroRNA profiles of iPSC lines were similar to each other and distinct from those of ESCs and mature cells

We examined miRNA expression patterns of three independent biological replicates of all four iPSC lines by direct detection of miRNA targets with the nCounter® miRNA expression assay. All samples passed all the QC parameters. We examined 798 miRNA probes, covering 827 mature miRNA sequences (21 probes detected > 1 similar miR sequences). Initial data processing was described above to yield normalized counts of each probe. Normalized counts of negative control probes had an average of 6.05 (S.D. = 12.7937, 95% CI [−19.03, 31.12]), so only miRNA probes with normalized counts above 31.12 were considered as expressed miRNAs in our dataset. With this cut-off, a total of 205 mature miRNAs were detected in at least one iPSC line, while 622 mature miRNAs were not detected in any iPSC lines (Table 2).

**Table 2:** Numbers of expressed miRNAs in each iPSC line

	Numbers of miRNAs (numbers of probes detected)			
	SF	SK	ST	SU
Expressed	145 (140)	151 (145)	163 (151)	152 (142)
Not expressed	682 (658)	676 (653)	664 (647)	675 (656)

**Fig. 2:**
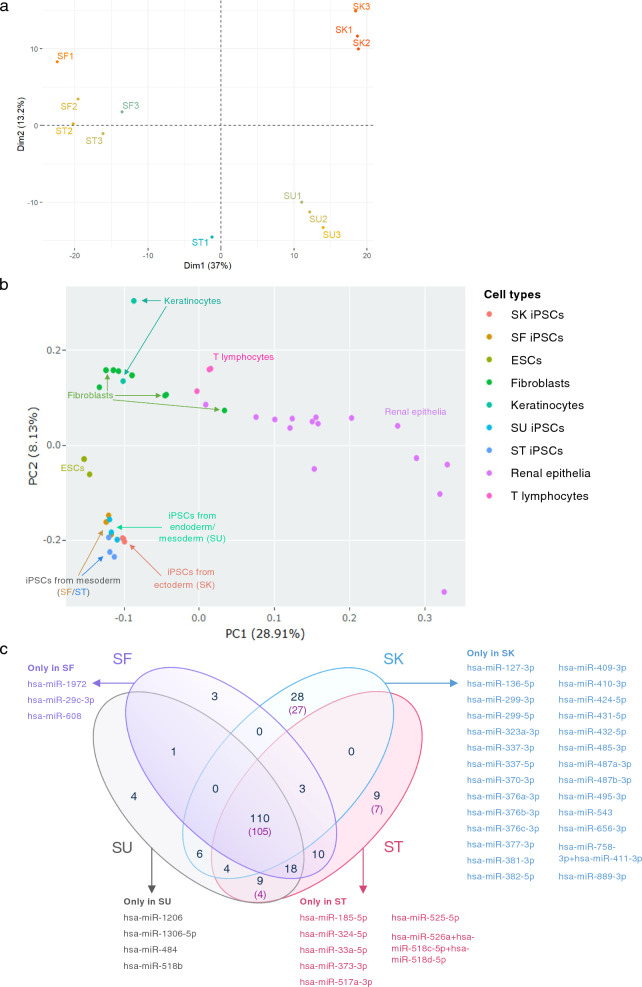
Normalized count numbers of the four iPSC lines and their analyses. The mean normalized count number of each probe was calculated from the normalized count numbers of the three biological replicates. **a** PCA of mean normalized count numbers of the SF, SK, ST, and SU iPSC lines. **b** PCA of NanoString^®^ count numbers of the four iPSC lines along with previously published NanoString^®^ count numbers of related cell types including embryonic stem cells (ESCs), fibroblasts, keratinocytes, renal epithelial cells, and T lymphocytes. **c** Venn diagram of microRNAs expressed in iPSC lines. Inside the Venn diagram, the numbers in black are the numbers of expressed mature miRNA sequences, while the numbers in purple in parentheses are the numbers of the nCounter^®^ miRNA expression assay probes that were used to detect those miRNAs (if different from the numbers of miRNAs)

We examined the miRNA count numbers of each cell line using PCA, and found that the triplicate cell lines clustered together and separated from triplicates of other iPSC lines, except for SF and ST which clustered closely together (Fig. 2a). We also examined relevant NanoString^®^ count numbers that were deposited in the Gene Expression Omnibus (GEO) database [36]. Deposited NanoString^®^ count numbers included were those of ESCs (GEO Accession numbers GSE26802 [37] and GSE81208 [38]) and mature cells from which these 4 iPSC lines of interest were derived from, including fibroblasts (GSE68185 [39], GSE84971 [40], GSE86801 [38], and GSE118888 [41]), keratinocytes (GSE69136 [42]), renal epithelial cells (GSE116251 [43]), and T lymphocytes (GSE57649 [44], GSE116880 [45], and GSE122943 [46]). As multiple datasets were analyzed, we employed the NanoStringNorm package for normalization to minimize any potential batch effects [27]. The miRNA profiles of the SF, SK, SU, and ST iPSC lines clustered together but away from those of ESCs. However, these stem cells, including ESCs and iPSCs, clustered nearby in the same quadrant of the PCA plot, while differentiated fibroblasts, keratinocytes, renal epithelial cells, and T lymphocytes scattered in other quadrants (Fig. 2b).

### Differentially expressed microRNAs and pathway analysis

**Fig. 3:**
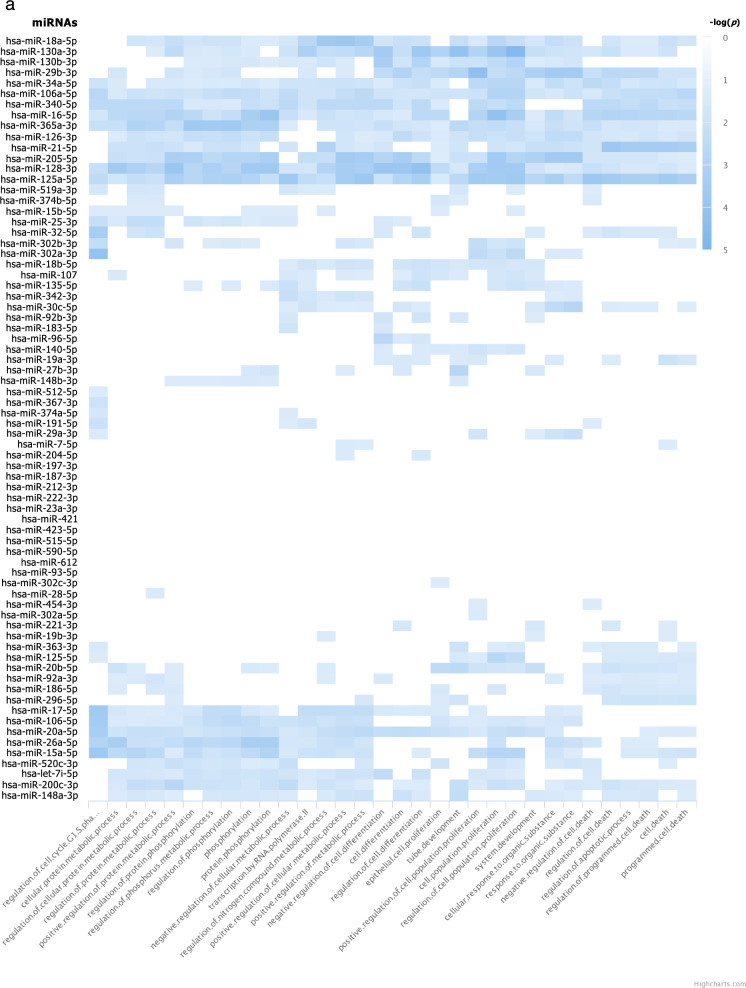

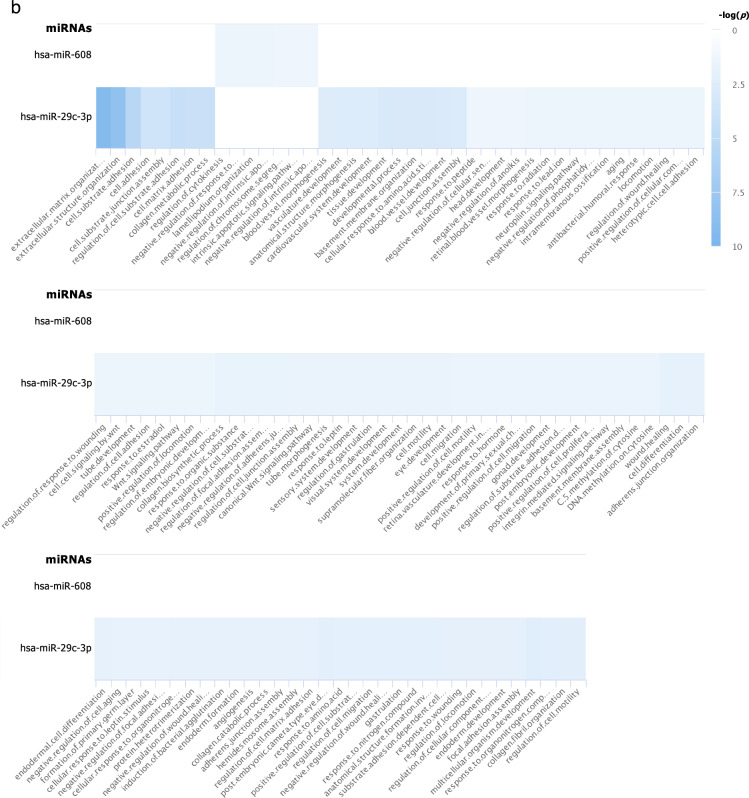

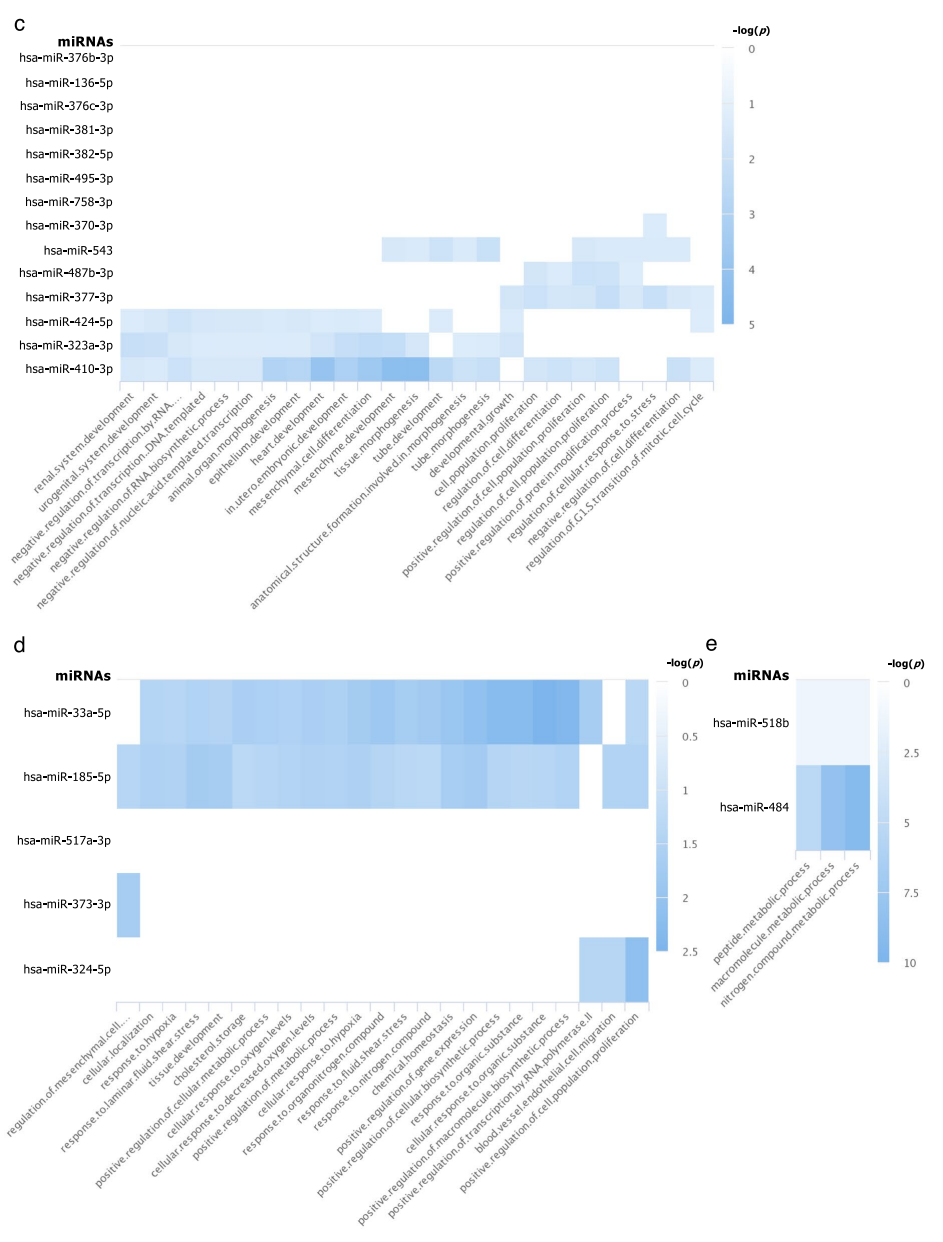

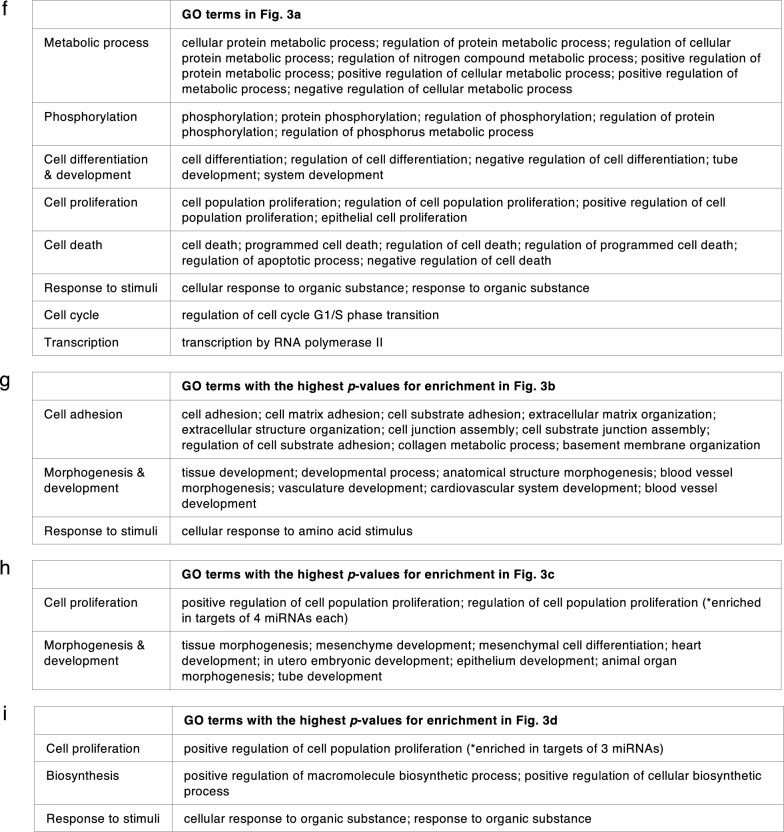
Pathway analyses: **a** Enriched pathways of targets of the 110 miRNAs commonly expressed in all 4 iPSC lines (intersect of all 4 circles in the Venn diagram in Fig. 2c); and enriched pathways of targets of uniquely expressed miRNAs in **b** the SF iPSC line, **c** the SK iPSC line, **d** the ST iPSC line, and **e** the SU iPSC line. Blue rectangles indicate that the targets of the miRNAs on the y-axis are significantly enriched for the biological pathways shown on the x-axis. The different shades of blue represent the -log_10_(*p-*values) of the enrichment result as specified on the right of each graph. The figures shown here were generated by miRPathDB v2.0 (https://mpd.bioinf.uni-sb.de/) [29] and slightly modified only to enhance legibility. All the biological processes in **a** and top biological processes in **b**, **c**, and **d** were separated into categories in **f**, **g**, **h**, and **i** respectively

From 205 mature miRNAs that were detected in at least one of the four iPSC lines, 110 miRNAs (which accounted for 67.5–75.9% of miRNAs expressed in each iPSC line) were common among all four iPSC lines (Fig. 2c). The top biological processes, those targeted by the greatest number of miRNAs, from enriched pathway analyses of these 110 miRNAs are involved in positive regulation of cell population proliferation, negative regulation of cell differentiation, and negative regulation of cell death (Fig. 3a, f). These results are as expected of PSCs that are in a proliferative state and are not undergoing differentiation.

Next, we examined miRNAs that were detected in only one iPSC line. Only 44 miRNAs belonged to this category – SF had 3 unique miRNAs (2.1%), SK had 28 unique miRNAs (18.5%), ST had 9 unique miRNAs (5.5%), and SU had 4 unique miRNAs (2.6%) (Fig. 2c).

The targets with strong experimental evidence of the three miRNAs that were uniquely expressed in SF, which included miR-1972, miR-29c-3p, and miR-608 (Fig. 2c), were enriched in 115 biological processes, but none were enriched by targets of more than one miRNA as analyzed by miRPathDB 2.0 algorithm [29] (Fig. 3b). Generally, enriched pathways are involved in a wide variety of processes which are not specific to fibroblast development (Fig. 3b, g). Although *hsa*-miR-29c-3p was also detected in mature fibroblasts in the GEO database [38–41], it does not appear to be a fibroblast-specific miRNA because miR-29-family miRNAs have been reported to be expressed in most tissue types and to function in a wide variety of cells, including osteoblasts, osteoclasts, fibroblasts, immune cells, adipose tissues, etc. [47, 48].

For 28 miRNAs that were uniquely expressed in SK, their targets were found to be enriched in more than 800 biological processes. When we analyzed the enriched pathways for targets of > 1 miRNAs, 165 biological processes were found. 25 pathways that were significantly enriched for targets of the most numbers of miRNAs were pathways involved in multicellular and anatomical structure development (Fig. 3c, h). Since these pathways are involved in differentiation of organs from all germ layers, it suggests that targets of unique miRNAs in SK are not specifically targeting genes in organ development lineage of only ectodermal germ layer. Therefore, they do not demonstrate restricted characteristics of its cell types of origin. This is consistent with the observation that all 28 miRNAs uniquely expressed in SK were not detected in keratinocytes deposited in the GEO database [42].

The target genes of 9 miRNAs uniquely expressed by ST were also enriched in more than 400 biological processes; 23 of which were targeted by > 1 miRNAs (Fig. 3d, i). Pathway analyses suggest that ST derived from peripheral T lymphocytes might possibly have some characteristics of their hematoendothelial lineage; however, these enriched pathways are more likely to be utilized by endothelial cells than by lymphocytes. Although *hsa*-miR-185-5p and *hsa*-miR-324-5p were also detected in normal T lymphocytes [44–46], they have been studied in a wide variety of conditions and pathways [49–53], and were not specific to peripheral blood’s T lymphocytes.

Finally, SU only had 4 unique miRNAs; however, there were no enriched pathways of targets with strong experimental evidence of any of these 4 miRNAs. 160 pathways were shown to be significantly enriched by targets with weak experimental evidence; the majority of which were enriched with potential targets of only one miRNA. Only 3 pathways were enriched by targets of > 1 miRNAs: peptide metabolic process, macromolecule metabolic process, and nitrogen compound metabolic process (Fig. 3e). One of the unique miRNAs in SU, *hsa*-miR-518b, was also expressed in normal renal epithelial cells [43]. However, previous studies reported that it was one of the placenta-associated miRNAs [54] with no obvious connections to renal cells or to epithelial cells in particular.

There was one previous study examining miRNA profiles of ESCs, cord blood-derived iPSCs and fibroblast-derived iPSCs; however they only focused on correlation analyses between ESCs and each iPSC line, so it was not possible to compare these with our results [10].

## Conclusion

In conclusion, miRNA profiles of the four iPSC lines showed that their overall epigenetic characteristics were similar to each other, albeit with few specific unique gene expression, rather than similar to each mature cell of origin or to embryonic stem cells.

## Limitations

Since this study was performed using one iPSC line from each cell type of origin, further experiments with the addition of more cell lines from the same cell types of origin may emphasize the conclusion of this study.

## Supplementary Information

Supplementary material 1.

## Data Availability

The NanoString reporter code count datasets generated during the current study are available in the NCBI GEO repository (GSE243742, https://www.ncbi.nlm.nih.gov/geo/query/acc.cgi?acc=GSE243742). The cell lines used in this study can be requested from the corresponding authors of the original research articles in which each cell line was generated [23–26].

## Abbreviations

ESC: Embryonic stem cell
GEO database: Gene Expression Omnibus database
iPSC: Induced pluripotent stem cell
miR, miRNA: MicroRNA
MTIs: MiRNA-target interactions
PCA: Principal component analysis
QC parameters: Quality control parameters
qRT-PCR: Quantitative real-time polymerase chain reaction
RCC: Reporter code counts
