# Supplementary material for: MicroRNA profiles of four induced pluripotent stem cell lines derived from distinct tissues
Source: BMC Res Notes. 2025 Aug 17;18:356. doi: 10.1186/s13104-025-07437-3 (PMC12358071; doi:10.1186/s13104-025-07437-3)
Supplement: Supplementary file 1 — Supplementary material 1. [file 13104_2025_7437_MOESM1_ESM.pdf]

Supplementary Materials

Supplementary Methods

miRNA expression profile analysis

miRNA expression profile of each iPSC line was examined by the nCounter® miRNA expression assay (Human v3 miRNA panel) on the nCounter® Analysis System instruments according to the manufacturer's protocol (NanoString Technologies). Briefly, 100 ng of total RNA were annealed and ligated to unique tags in the nCounter® miRNA Tag Reagent. Purified miRNA-tag pairs were then hybridized to Reporter and Capture probes overnight. Then, target/probe complexes were washed, purified, and immobilized on the sample cartridge on the nCounter® Prep Station instrument. Finally, digital images of the sample cartridge were processed and reporter code counts (RCC) were performed by the nCounter® Digital Analyzer instrument. RCC files were exported from the instrument for further analysis as described in the main manuscript.

Normalization of NanoString miRNA expression data from Gene Expression Omnibus

NanoString reporter code counts were retrieved from the Gene Expression Omnibus (GEO) database. NanoString RCC data from distinct GEO data series were normalized by the NanoStringNorm package in the R statistical language [27] before PCA was performed. Briefly, normalization was performed following these steps: code-count normalization with geometric mean of positive control counts, background correction by subtracting the mean of negative control counts plus two standard deviations, and sample content normalization with geometric mean of housekeeping gene counts [27].

Supplementary Results

miRNA expression assay counts: quality checks and intra-group variations

We examined miRNA expression patterns of three independent biological replicates of four iPSC lines by direct detection of miRNA targets with the nCounter® miRNA expression assay. First, none of the samples were flagged for any abnormality in any QC parameters. Every

replicate contained at least 3.95 x 10^5 raw counts. Second, we assessed intra-group variation by agglomerative clustering and regression analysis of normalized counts of each biological replicate. These analyses demonstrated that all intra-group replicates are more similar to each other than to samples from other iPSC lines, suggesting no significant intra-group variations (Fig. S3a-b).

Supplementary Fig. S1

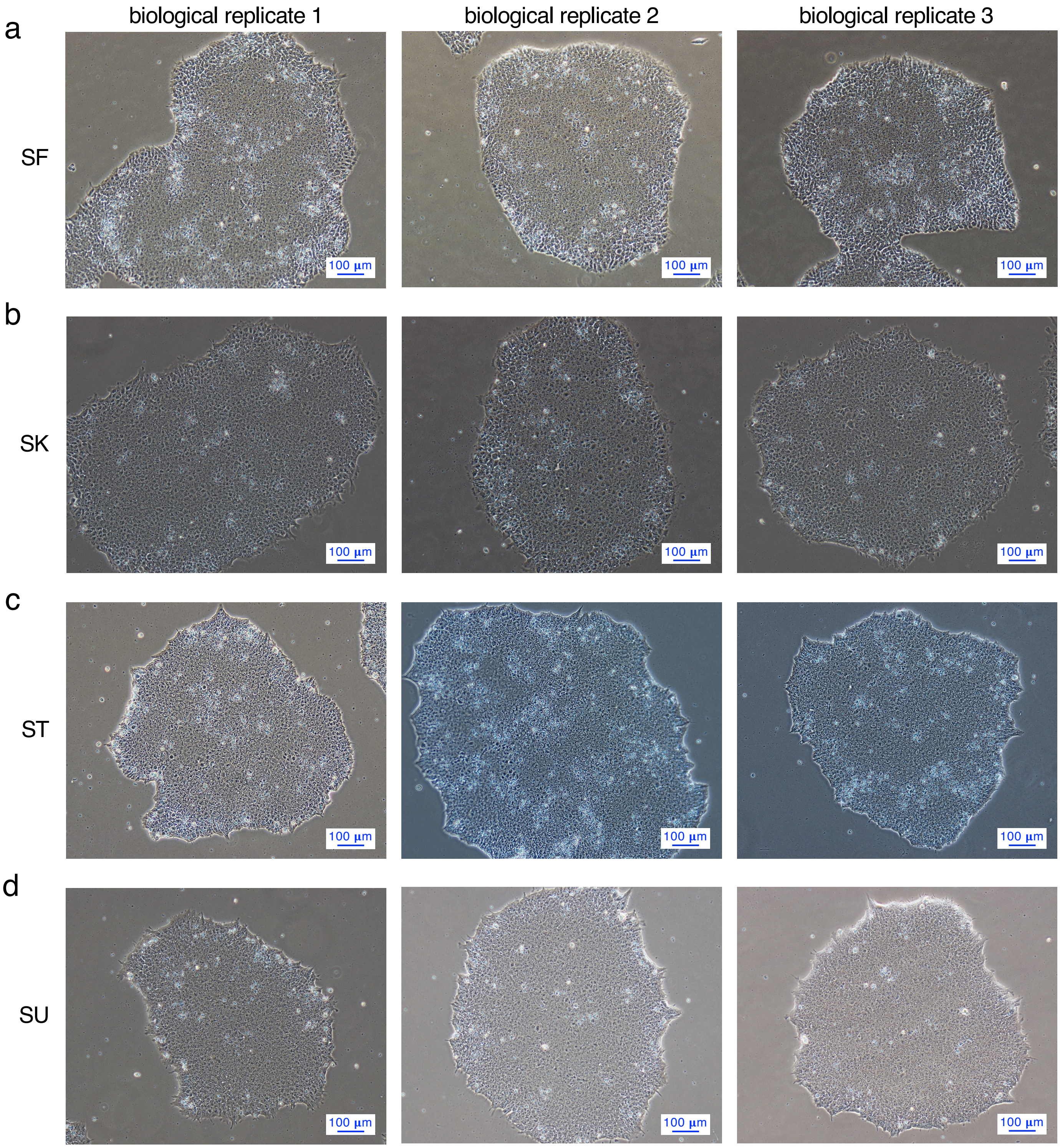

Supplementary Fig. S1 Morphology of iPSCs on the day of sample collection for RNA extraction. Figures shown here are triplicates of these iPSC lines: (**a**) SF, (**b**) SK, (**c**) ST, and (**d**) SU. All figures were taken at the same magnification.

Supplementary Fig. S2

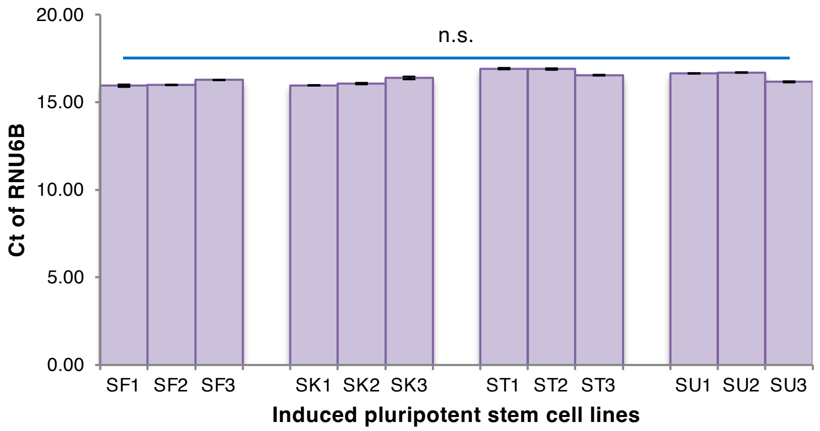

Supplementary Fig. S2 Expression levels of RNU6B, a housekeeper small RNA, in all iPSC samples**.** Average Ct values of technical replicates are shown as bar graph, along with their standard deviations as error bars. n.s. designates *p*-values > 0.05, as analyzed by independent-samples Kruskal-Wallis Test.

Supplementary Fig. S3

a

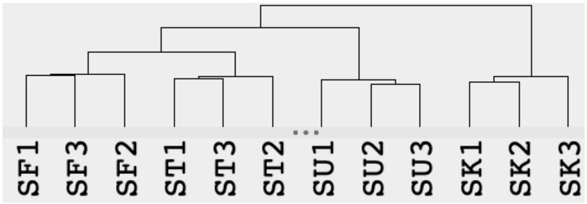

b

	SF1	SF2	SF3	SK1	SK2	SK3	ST1	ST2	ST3	SU1	SU2	SU3
SF1	1.0	0.88	0.88	0.73	0.74	0.71	0.86	0.87	0.87	0.84	0.84	0.83
SF2	0.88	1.0	0.90	0.71	0.71	0.70	0.87	0.86	0.88	0.84	0.84	0.82
SF3	0.88	0.90	1.0	0.72	0.72	0.70	0.89	0.88	0.89	0.85	0.85	0.84
SK1	0.73	0.71	0.72	1.0	0.93	0.92	0.71	0.71	0.72	0.75	0.75	0.74
SK2	0.74	0.71	0.72	0.93	1.0	0.91	0.70	0.71	0.72	0.74	0.74	0.75
SK3	0.71	0.70	0.70	0.92	0.91	1.0	0.69	0.71	0.70	0.74	0.74	0.74
ST1	0.86	0.87	0.89	0.71	0.70	0.69	1.0	0.89	0.89	0.84	0.84	0.83
ST2	0.87	0.86	0.88	0.71	0.71	0.71	0.89	1.0	0.89	0.86	0.84	0.82
ST3	0.87	0.88	0.89	0.72	0.72	0.70	0.89	0.89	1.0	0.85	0.86	0.83
SU1	0.84	0.84	0.85	0.75	0.74	0.74	0.84	0.86	0.85	1.0	0.91	0.89
SU2	0.84	0.84	0.85	0.75	0.74	0.74	0.84	0.84	0.86	0.91	1.0	0.90
SU3	0.83	0.82	0.84	0.74	0.75	0.74	0.83	0.82	0.83	0.89	0.90	1.0

Supplementary Fig. S3 a. A dendrogram tree generated from agglomerative clustering of normalized counts of each biological replicate (designated as 1, 2, and 3; n = 3 biological replicates) of SF, SK, ST, and SU iPSC lines, as generated by nSolver™ 4.0 Analysis Software **b**. R-squared values from linear regression analyses of all pairwise comparisons between each biological replicate of the SF, SK, ST, and SU iPSC lines
